# Neoadjuvant imatinib in patients with locally advanced non metastatic GIST in the prospective BFR14 trial

**DOI:** 10.1186/1471-2407-11-72

**Published:** 2011-02-15

**Authors:** Aurore Blesius, Philippe A Cassier, François Bertucci, Jerome Fayette, Isabelle Ray-Coquard, Binh Bui, Antoine Adenis, Maria Rios, Didier Cupissol, David Pérol, Jean-Yves Blay, Axel Le Cesne

**Affiliations:** 1 Department of medicine, Institut Gustave Roussy, 39 bis rue Camille Desmoulins, 94800 Villejuif, France; 2 Department of medicine, Centre Léon Bérard, 28 rue Laennec, 69008 Lyon, France; 3 Department of medicine, Institut Paoli-Calmettes, 232 boulevard Sainte Marguerite, 13009 Marseille, France; 4 Department of medicine, Institut Bergonié, 229 cours Argonne, 33000 Bordeaux, France; 5 Department of medicine, Centre Oscar Lambret, 3 rue Frederic Combemale, 59000 Lille, France; 6 Department of medicine, Centre Alexis Vautrin, 6 avenue Bourgogne, 54500 Vandoeuvre-lès-Nancy, France; 7 Department of medicine, Centre Val d'Aurelle, 31 rue Croix Verte, 34000 Montpellier, France; 8 Department of biostatistics, Centre Léon érard, 28 rue Laennec, 69008 Lyon, France

## Abstract

**Background:**

The role of surgery in the management of patients with advanced gastrointestinal stromal tumors (GIST) in the era of imatinib mesylate (IM) remains debated. We analyzed the outcome of patients with non metastatic locally advanced primary GIST treated with IM within the prospective BFR14 phase III trial.

**Methods:**

The database of the BFR14 trial was searched for patients with no metastasis at time of inclusion. Patients treated for recurrent disease were excluded. Twenty-five of 434 patients met these criteria.

**Results:**

Fifteen of 25 patients (60%) had a partial response to IM. Nine of the 25 patients (36%) underwent surgical resection of their primary tumor after a median of 7.3 months of IM treatment (range 3.4-12.0). Per protocol patients received continuous IM treatment in the post resection period, in an adjuvant setting. With a median follow-up of 53.5 months, there was a significant improvement in progression-free survival (PFS) and overall survival (OS) for patients who underwent surgical resection *versus *those who did not (median not reached *vs *23.6 months, p = 0.0318 for PFS and median not reached *vs *42.2 months, p = 0.0217 for OS). In the group of patients who underwent resection followed by IM, the 3-year PFS and OS rates were 67% and 89% respectively

**Conclusions:**

Following neoadjuvant IM for non metastatic locally advanced GIST 9 of 25 patients (36%) were selected for resection of the primary tumor. OS and PFS figures were close to those of localised intermediate or high risk GIST (70% at 5 years) in the subgroup of operated patients, while the outcome of the non-operated subgroup was similar to that of metastatic GIST.

## Background

Gastrointestinal stromal tumors (GIST) are the most frequent mesenchymal tumors of the gastrointestinal tract and are thought to originate from the interstitial cells of Cajal [1]. The management of advanced and metastatic GIST has considerably improved with the use of imatinib mesylate (IM). Approximately 50 to 70% of unselected patients with advanced or metastatic GIST respond to IM and the median progression-free survival (PFS) is 20 to 24 months [1,2] in a highly chemo-resistant disease [3]. IM, originally designed as a specific inhibitor of the Bcr-Abl kinase for the treatment of chronic myelogenous leukaemia, was also shown to be a potent inhibitor of the tyrosine kinase activities of KIT, PDGFR and CSF1R. *KIT *or *PDGFRA *mutations are considered an early event in the oncogenesis of GIST [4,5] and are found in roughly 90% of cases. IM is considered a standard of care for patients with advanced disease and as adjuvant therapy for completely resected localised GIST [6-8]; however, its role in the neoadjuvant setting is currently under investigation.

The outcome of patients with locally advanced and unresectable GIST is generally considered to be similar to that of patients with metastatic disease. Although first-line treatment with IM produces high rates of disease control in patients with advanced disease, most patients experience disease progression due to the emergence of molecularly resistant clones within 2-3 years after treatment initiation. This observation led several authors to investigate the value of surgical excision of residual disease following response to IM, before the development of secondary resistance. Several publications have reported on the feasibility of surgery following primary treatment with IM [9-14], but little is known about the exact benefit in terms of progression-free or overall survival. Furthermore, all these studies included patients with both locally advanced and metastatic disease. All were retrospective, except the recently reported RTOG-0132 study [11].

We report here the retrospective analysis of patients with locally advanced non metastatic GIST who received primary medical therapy with IM in the BFR14 prospective trial [15], with special attention to the patients who underwent secondary surgery of their primary tumor.

## Methods

### BFR14 population

The BFR14 trial is a phase III trial randomizing interruption *versus *continuation of imatinib beyond one year of treatment for non progressive patients [15]. After the results of the randomisation at one year were known, the protocol was amended to allow randomisation after three years of treatment, and more recently after five years of treatment [16].

Inclusion criteria were: age at least 18 years, histological confirmation of locally advanced and/or metastatic GIST, immunohistochemical documentation of c-KIT (CD117) expression, and Eastern Cooperative Oncology Group performance status of 0 to 3. Patients had to be previously untreated with imatinib, with no history of previous malignancy, and were required to have normal renal, cardiac, and hepatic functions. No concurrent anticancer therapy was allowed. All patients gave written informed consent before inclusion.

Imatinib was given orally at 400 mg per day, as a single daily dosing. Clinical and biological tolerance was assessed weekly during the first month of treatment, every 2 weeks the following month, then monthly for three months, and every three months thereafter. Initial assessment included a complete history, clinical examination, serum biochemistry, liver function test, whole blood count and computed tomography scan (CT scan) and/or magnetic resonance imaging (MRI) of the tumor. Imaging techniques were repeated after 6 and 12 weeks of treatment and every 3 month thereafter. Response was graded according to the RECIST criteria [17].

### Definition of non metastatic locally advanced GIST

To be eligible for the present substudy, patients of the BFR14 study were to have primary non metastatic GIST as assessed by the local multidisciplinary team at each participating site, and no prior surgery, thus excluding patients with recurrent GIST.

### Statistical analysis

Data were described using the median and range for continuous variables and using percentages with 95% confidence intervals for qualitative variables. Comparisons were performed using the chi-square test, Fisher's exact test, or Wilcoxon's rank-sum test as appropriate. Survival times were calculated from the date of entry in the BFR14 trial (i.e. initiation of IM treatment) and were displayed using the Kaplan-Meier method [18]. Progression-free survival (PFS) was defined as the time from the date of inclusion (start of IM) to the date of progression on IM 400 mg/day or death. Overall survival (OS) was defined as the time from the date of inclusion to death of any cause. Differences in survival distributions were tested using the Log-Rank test. Differences were considered statistically significant when p ≤ 0.05. Statistical analyses were performed using the SPSS 12.0.1 (SPSS Inc, Chicago, IL) software package.

## Results

As of April 2010, 434 patients have been included in the BFR14 trial. Sixty patients were registered as having no known metastasis initially. Of these 60 patients, only 25 had no previous history of surgery for GIST and were included in the present substudy. The 35 remaining cases included patients with locally advanced disease who received IM as additional therapy following R2 resection or patients with locally advanced recurrent disease.

**Figure 1:**
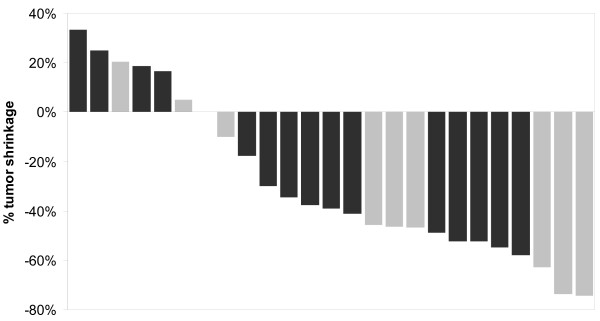
**Waterfall plot of patients' best response**. Dark grey bars represent patients who did not undergo resection of their primary tumor. Light grey bars represent patient who had their primary tumor resected.

Characteristics of these 25 patients and response to IM are presented in Table 1. Median age was 65.5 (range 39.8-80.5) years, 16 patients were males and the median performance status was 1 (range 0-3). Median tumor size at baseline was 15 cm. No complete response was seen following treatment with IM, 15 patients (60%) had a partial response (PR) after a median of 4.0 (range 1.4-12.8) months, 7 patients (28%) had stable disease (SD) as their best response, while 3 (12%) patients had progressive disease (PD) as their best response (Figure 1).

**Table 1:** Patients' main characteristics

	All		Patients who underwent surgery		Patients who were operated	
Characteristics	N	%	N	%	N	%
	25		9		16	
Age						
Median (range)	65.5 (39.8-80.5)		60.0 (39.8-80.5)		69.7 (40.7-81.9)	
Gender						
Male	16	64%	6	67%	10	63%
Female	9	36%	3	33%	6	38%
Tumor location						0%
Stomach	4	16%	1	11%	3	19%
Small intestine	7	28%	4	44%	3	19%
Peritoneum	7	28%	1	11%	6	38%
Oesophagus	2	8%	0	0%	2	13%
Rectum	4	16%	3	33%	1	6%
Pelvis	1	4%	0	0%	1	6%
Tumor size (mm)						
Median (range)	150	(36-280)	150	(45-280)	149	(36-200)
≤ 50	2	8%	1	11%	1	6%
50 < - ≤ 100	7	28%	3	33%	4	25%
> 100	16	64%	5	56%	11	69%
WHO PS						0%
0	10	40%	4	44%	6	38%
1	10	40%	5	56%	5	31%
2	2	8%	0	0%	2	13%
3	1	4%	0	0%	1	6%
Not reported	2	8%	0	0%	2	13%
Best response to IM						
PR	15	60%	6	67%	9	56%
SD	7	28%	2	22%	5	31%
PD	3	12%	1	11%	2	13%

Nine of the 25 patients (38%) underwent surgery after a median of 7.3 (range 3.4-12.0) months of IM treatment. Surgery was performed by the local surgeons who had initially refuted operation. In 4 patients tumor shrinkage with IM allowed resection at the price of a less morbid operation. In 2 patients surgical resection was performed as a salvage procedure for disease progressing on IM (primary progession in one case and progression following initial response in another case). The reason(s) that lead to the decision to operate each patient are listed in table 2. Patients who underwent surgery tended to be younger (median 60.0 *vs *69.7, p = 0.174) and have better performance status (PS) (PS 0-1 9/9 *vs *11/16, p = 0.253) than the non-operated patients, although these differences were not statistically different (table 1). The sex ratio was similar between the two groups. Non-operated patients were more likely to have GIST from the peritoneum or the oesophagus (7/16 *vs *1/9, p = 0.040). Baseline tumor size was comparable between patients who were operated and those who were not (p = 0.671, Mann and Whitney test). The response rate was slightly superior in the operated group (67% *vs *56% in the non-operated group), but the difference was not statistically significant (p = 0.691). The clinical benefit rate (PR+SD) was similar in the two groups (89% and 88%, p = 1.0). Among the 9 patients who underwent surgery, 6 had a partial response after a median of 4.2 months of treatment with IM, and surgery was microscopically complete (R0) for 5 of them and macroscopically incomplete in one of them (R2). Two patients underwent surgery with a volumetric response which was less than a partial response, i.e. disease stabilization according to RECIST; and surgery was complete for both of them (R0). One patient underwent surgery while his disease was progressing according to RECIST: surgical excision was macroscopically complete but with positive margins, and this patient remains in complete response (CR) 66.4 months after the procedure despite his refusal to continue IM post-operatively. The other eight operated patients accepted to continue IM after surgery as specified by the study protocol. Three of 9 patients progressed 13.3, 17.0 and 24.8 months after surgery; the other 6 patients are currently in CR at a median of 55.4 (range 50.0-66.5) months after surgery.

**Table 2:** Reasons for operation following treatment with Imatinib

Patient N	Age at surgery	Tumor location	Best % tumor shrikage	Time to surgery	reason for operation
4	43	Rectum	-47	12,1	Significant response after 12 months on imatinib, enabling tumor resection
6	66	Mesentery	-63	6,7	Large tumor lesion, decision to operate following tumor shrinkage on imatinib
7	81	Rectum	-47	8,1	After initial response, patient had early signs of progression (increased blood flow on DCE-ultrasound) and was therefore operated before actual RECIST progression
8	40	Rectum	-46	7,3	Surgery enabled following tumor shrinkage
12	43	Small bowel	-74	6,5	Surgery planned prior to treatment with imatinib (true neoadjuvant)
13	61	Small bowel	-74	11,7	Surgery enabled following tumor shrinkage
14	71	Small bowel	20	3,4	Rapid progression on imatinib 400 mg/d, dose increased to 600 mg/d which was poorly tolerated, salvage surgery seemed feasible. Resection was R1
15	76	Stomach	5	4,4	No response on imatinib with poor tolerance. Following surgery this patient was restarted on a lower dose of IM.
16	50	Small bowel	-19	7,3	Stable disease after 6 months on imatinib, surgery was deemed feasible by surgeon.

**Figure 2:**
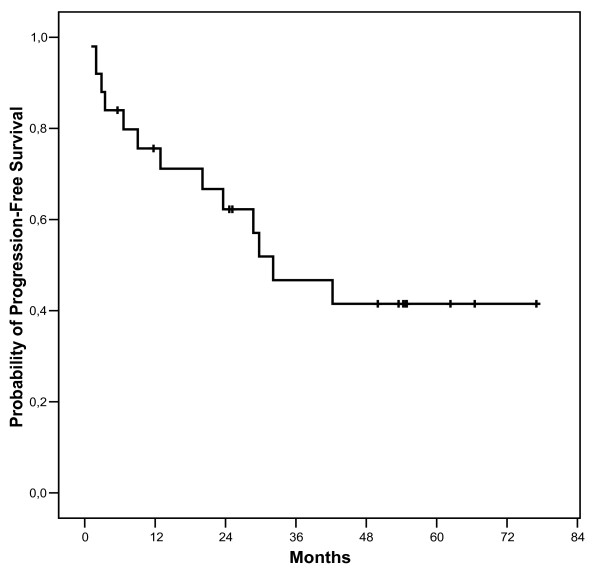
**Progression-free-survival**.

**Figure 3:**
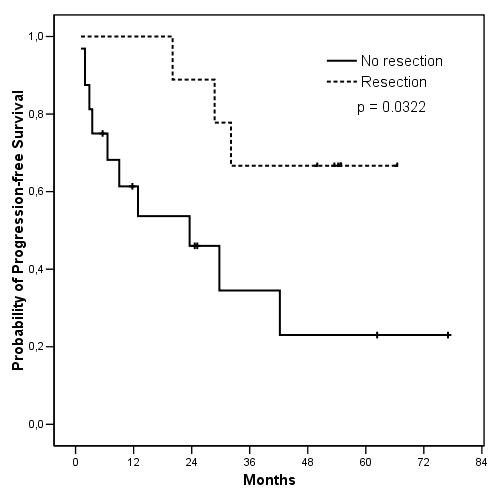
**Progression-free survival according to surgical status**.

**Figure 4:**
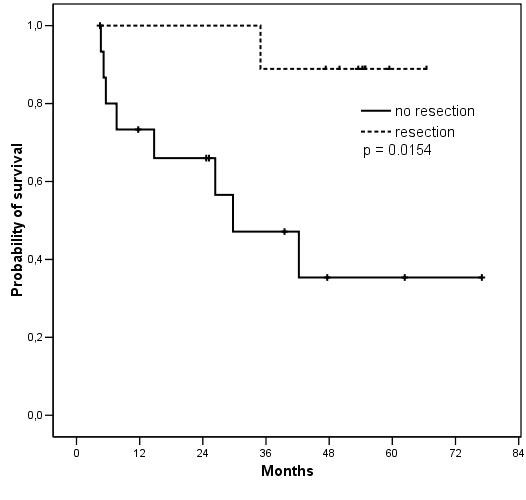
**Overall survival according to surgical status**.

With a median follow-up of 53.5 (range 4.4-77.0) months, the median PFS is 32.1 months for the 25 patients (Figure 2), while median OS is not reached (not shown). PFS was significantly longer (figure 3) for the 9 patients who underwent surgery after IM than for the 16 patients who continued medical treatment alone (median PFS not reached *vs *23.6 months respectively, p = 0.0322). Similarly, OS (figure 4) was longer for the resected group (median OS not reached *vs *29.7 months respectively, p = 0.0154). This difference in PFS and OS persisted when analysis was limited to patients with PR or SD on IM, after exclusion of progressive patients, although the difference in PFS did not reach statistical significance in this analysis (median 29.7 vs not reached, p = 0.0998 and 42.2 vs not reached, p = 0.0333 for PFS and OS respectively). When analysing only the 15 patients who had PR to IM, the median PFS was 29.7 months for non-resected patients *vs *not reached for resected patients (p = 0.2829).

## Discussion

Imatinib mesylate is the new paradigm of treatment targeting the initial causal molecular event in solid tumors. However, it is still unclear whether patients with locally advanced or metastatic disease are cured, as shown by the results of the BFR14, which showed consistent disease progression following treatment interruption after 1, 3 and 5 years of treatment [15,16]. The role of surgery during or following treatment with IM for locally advanced disease remains controversial. Several expert centers throughout the world have reported their results with surgical resection following treatment with IM for both locally advanced and metastatic GIST[9-14]. All these studies are unicentric and retrospective. Most of them have underlined the strong correlation between the disease status regarding response to IM before surgery and the outcome after surgery. Patient with multifocal progressive disease at the time of surgery have a short PFS following surgery (3-6 months), while patients with unifocal disease progression have a 6 months median PFS with some patients remaining free of progression after long-term follow-up [12,13]. The recently reported RTOG0132/ACRIN6665 trial [11] is the first study to prospectively assess the role of preoperative IM (during 8 to 12 weeks) in patients with primary locally advanced (≥ 5 cm: 30 patients) and/or metastatic/recurrent (≥ 2 cm: 20 patients) GIST. In this study, toxicity was minimal and did not modify post-operative morbidity. However, because it was a single arm phase II trial, this study did not answer the question of the benefit of surgery in patients with locally advanced initially inoperable GIST.

Our present study is the first multi-centric series to address the issue of benefit of surgery after neoadjuvant IM in this setting. We show that among 25 patients with non-metastatic locally advanced GIST, 9 patients (36%) were selected to undergo surgical resection following primary medical treatment with IM. These 9 patients had improved PFS and OS compared to non-operated patients, with survival rates close to those observed for localised intermediate or high risk GIST, whereas survival of non-operated patients was similar to that of patients with metastatic disease. Although these results suggest an improved outcome for operated patients, this study has some obvious limitations. One of these limitation is that patients were selected and not randomised to undergo surgery and were therefore more likely to benefit from the procedure based on medical judgement by the investigators at each site. Furthermore, our series is small and retrospective, precluding any definitive conclusion. As previously mentioned our observation is likely biased since selection of patients for surgery may be linked to other prognostic factors such as tumor location, patient's age, performance status as reflected by the differences (though not significant) seen in our series between the operated and non-operated groups. The response to IM may be another source of bias as more patients had a PR in the operated group than in the non-operated group. However, survival remained better in the operated group even when considering only patients with partial response or patients with clinical benefit (PR or SD). A possible source of difference of survival between the two groups may be the randomisation (see the "patients and methods" section). Six of 25 patients were randomised, two in the IM continuation arm and four in the interruption arm. All of the four patients randomised to interruption were in the non surgical group, therefore introducing a bias. However, PFS and OS were still significantly better in the surgery group when these four cases were removed from analysis (9.0 months *vs *median not reached p = 0.0037 and 26.3 months *vs *median not reached, p = 0.0128 respectively for PFS and OS).

Another bias source of this multicentric study lies in the inclusion criterion of initial unresectability, which was left at the treating physician's discretion. Therefore, some patients may have had truly unresectable disease, while others may have had disease that was actually resectable at the price of a major procedure, in which case primary medical treatment appeared to be the best option. Resectability, before and after IM, was assessed by multidisciplinary teams, including surgeons expert in GIST management.

## Conclusions

Overall, this and other reports can not lead to any definitive conclusion regarding the benefit of surgery in patients with locally advanced GIST treated with IM. This benefit can only be demonstrated in randomised prospective trials, which are ongoing in the metastatic setting. However, since patients with locally advanced disease who become operable following IM appear to benefit from resection of the primary tumor, we think that surgery should be proposed, or at least discussed, in this subgroup of patients when the disease no longer responds to IM.

## Competing interests

The authors declare that they have no competing interests.

## Authors' contributions

AB contributed to provision of patients, participated in data acquisition, analysed the data, and helped draft the manuscript; PAC designed the study, contributed to patient provision, participated in data acquisition, analysed the data, and drafted the manuscript; FB contributed to patient provision and helped draft the manuscript; JF designed the study and contributed to patient provision; IRC and BB, AA contributed to patient provision and helped draft the manuscript, MR and DC contributed to provision of patients; DP contributed to the design of the BFR14 study and participated in data acquisition; JYB and AL contributed to the design of the BFR14 study, to patient provision and helped draft the manuscript. All authors have read and approved the final manuscript.

## Pre-publication history

The pre-publication history for this paper can be accessed here:

http://www.biomedcentral.com/1471-2407/11/72/prepub

## References

[B1] Demetri GD von MM Blanke CD Van den Abbeele AD Eisenberg B Roberts PJ Efficacy and safety of imatinib mesylate in advanced gastrointestinal stromal tumors N Engl J Med 2002 347 472 480 10.1056/NEJMoa020461 12181401

[B2] Verweij J Casali PG Zalcberg J Lecesne A Reichardt P Blay JY Progression-free survival in gastrointestinal stromal tumours with high-dose imatinib: randomised trial Lancet 2004 364 1127 1134 10.1016/S0140-6736(04)17098-0 15451219

[B3] Cassier PA Dufresne A Arifi S El SH Labidi I Ray-Coquard I Imatinib mesilate for the treatment of gastrointestinal stromal tumour Expert Opin Pharmacother 2008 9 1211 1222 10.1517/14656566.9.7.1211 18422477

[B4] Hirota S Isozaki K Moriyama Y Hashimoto K Nishida T Ishiguro S Gain-of-function mutations of c-kit in human gastrointestinal stromal tumors Science 1998 279 577 580 10.1126/science.279.5350.577 9438854

[B5] Heinrich MC Corless CL Duensing A McGreevey L Chen CJ Joseph N PDGFRA activating mutations in gastrointestinal stromal tumors Science 2003 299 708 710 10.1126/science.1079666 12522257

[B6] Demetri GD Benjamin RS Blanke CD Blay JY Casali P Choi H NCCN Task Force report: management of patients with gastrointestinal stromal tumor (GIST)--update of the NCCN clinical practice guidelines J Natl Compr Canc Netw 2007 5 Suppl 2 S1 29 quiz S30 17624289

[B7] Casali PG Jost L Reichardt P Schlemmer M Blay JY Gastrointestinal stromal tumors: ESMO clinical recommendations for diagnosis, treatment and follow-up Ann Oncol 2008 19 Suppl 2 ii35 ii38 10.1093/annonc/mdn080 18456761

[B8] Dematteo RP Ballman KV Antonescu CR Maki RG Pisters PW Demetri GD Adjuvant imatinib mesylate after resection of localised, primary gastrointestinal stromal tumour: a randomised, double-blind, placebo-controlled trial Lancet 2009 373 1097 1104 10.1016/S0140-6736(09)60500-6 19303137 PMC2915459

[B9] Andtbacka RH Ng CS Scaife CL Cormier JN Hunt KK Pisters PW Surgical resection of gastrointestinal stromal tumors after treatment with imatinib Ann Surg Oncol 2007 14 14 24 10.1245/s10434-006-9034-8 17072676

[B10] Bonvalot S Eldweny H Pechoux CL Vanel D Terrier P Cavalcanti A Impact of surgery on advanced gastrointestinal stromal tumors (GIST) in the imatinib era Ann Surg Oncol 2006 13 1596 1603 10.1245/s10434-006-9047-3 16957966

[B11] Eisenberg BL Harris J Blanke CD Demetri GD Heinrich MC Watson JC Phase II trial of neoadjuvant/adjuvant imatinib mesylate (IM) for advanced primary and metastatic/recurrent operable gastrointestinal stromal tumor (GIST): early results of RTOG 0132/ACRIN 6665 J Surg Oncol 2009 99 42 47 10.1002/jso.21160 18942073 PMC2606912

[B12] Gronchi A Fiore M Miselli F Lagonigro MS Coco P Messina A Surgery of residual disease following molecular-targeted therapy with imatinib mesylate in advanced/metastatic GIST Ann Surg 2007 245 341 346 10.1097/01.sla.0000242710.36384.1b 17435538 PMC1877023

[B13] Raut CP Posner M Desai J Morgan JA George S Zahrieh D Surgical management of advanced gastrointestinal stromal tumors after treatment with targeted systemic therapy using kinase inhibitors J Clin Oncol 2006 24 2325 2331 10.1200/JCO.2005.05.3439 16710031

[B14] Rutkowski P Nowecki Z Nyckowski P Dziewirski W Grzesiakowska U Nasierowska-Guttmejer A Surgical treatment of patients with initially inoperable and/or metastatic gastrointestinal stromal tumors (GIST) during therapy with imatinib mesylate J Surg Oncol 2006 93 304 311 10.1002/jso.20466 16496358

[B15] Blay JY Le CA Ray-Coquard I Bui B Duffaud F Delbaldo C Prospective multicentric randomized phase III study of imatinib in patients with advanced gastrointestinal stromal tumors comparing interruption versus continuation of treatment beyond 1 year: the French Sarcoma Group J Clin Oncol 2007 25 1107 1113 10.1200/JCO.2006.09.0183 17369574

[B16] Le Cesne A Ray-Coquard I Bui B Rios M Adenis A Bertucci F Continuous versus interruption of imatinib (IM) in responding patients with advanced GIST after three years of treatment: A prospective randomized phase III trial of the French Sarcoma Group J Clin Oncol (Meeting Abstracts) 2007 25 10005

[B17] Therasse P Arbuck SG Eisenhauer EA Wanders J Kaplan RS Rubinstein L New guidelines to evaluate the response to treatment in solid tumors. European Organization for Research and Treatment of Cancer, National Cancer Institute of the United States, National Cancer Institute of Canada J Natl Cancer Inst 2000 92 205 216 10.1093/jnci/92.3.205 10655437

[B18] Kaplan EL Meier P Nonparametric estimation from incomplete observations J Am Stat Assoc 1958 53 457 481 10.2307/2281868

